# Integrating Caregiver Education and Support into Multidisciplinary Video Visits

**DOI:** 10.1093/geroni/igaa057.2286

**Published:** 2020-12-16

**Authors:** Michelle Rossi, Lauren Jost, Ina Engel, Carol Dolbee, Keisha Ward

**Affiliations:** 1 VA Pittsburgh Healthcare System, Pittsburgh, Pennsylvania, United States; 2 VA Pittsburgh Healthcare System, pittsburgh, Pennsylvania, United States; 3 University of Pittsburgh, pittsburgh, Pennsylvania, United States

## Abstract

The TeleDementia Clinic is an interdisciplinary longitudinal telehealth dementia clinic providing care to rural Veterans in Western Pennsylvania with cognitive decline. The TeleDementia Caregiver Support group uses telehealth to provide caregiver support and education to those caregivers with the highest levels of caregiver burden in this population. The support group has caregivers participating in the session at different rural clinics while a multidisciplinary team of professional (geropsychologist, geriatrician, nurse practitioner and others) are located at an urban VA medical center. All can interact via video telehealth connection. Each session provides a short educational session on caregiving topics that then provides a springboard for caregiver discussion about their own experiences. The multidisciplinary clinician team lend their expertise to the education and support of caregivers. Both quantitative and qualitative analysis of effectiveness of this model will be discussed.

